# Undergraduate student drinking and related harms at an Australian university: web-based survey of a large random sample

**DOI:** 10.1186/1471-2458-12-37

**Published:** 2012-01-16

**Authors:** Jonathan Hallett, Peter M Howat, Bruce R Maycock, Alexandra McManus, Kypros Kypri, Satvinder S Dhaliwal

**Affiliations:** 1 Western Australian Centre for Health Promotion Research, Curtin Health Innovation Research Institute, Curtin University, Kent Street, Bentley, Australia; 2 Centre for Behavioural Research in Cancer Control, Curtin University, 10 Selby Street, Shenton Park, Subiaco, Australia; 3 National Drug Institute, Curtin University, 10 Selby Street, Shenton Park, Subiaco, Australia; 4 Curtin Health Innovation Research Institute, Curtin University, Kent Street, Bentley, Australia; 5 School of Medicine and Public Health, University of Newcastle, University Drive, Callaghan, Australia; 6 Injury Prevention Research Unit, Department of Preventive and Social Medicine, University of Otago, 18 Frederick Street, Dunedin, New Zealand; 7 School of Public Health, Curtin University, Kent Street, Bentley, Australia

## Abstract

**Background:**

There is considerable interest in university student hazardous drinking among the media and policy makers. However there have been no population-based studies in Australia to date. We sought to estimate the prevalence and correlates of hazardous drinking and secondhand effects among undergraduates at a Western Australian university.

**Method:**

We invited 13,000 randomly selected undergraduate students from a commuter university in Australia to participate in an online survey of university drinking. Responses were received from 7,237 students (56%), who served as participants in this study.

**Results:**

Ninety percent had consumed alcohol in the last 12 months and 34% met criteria for hazardous drinking (AUDIT score ≥ 8 and greater than 6 standard drinks in one sitting in the previous month). Men and Australian/New Zealand residents had significantly increased odds (OR: 2.1; 95% CI: 1.9-2.3; OR: 5.2; 95% CI: 4.4-6.2) of being categorised as dependent (AUDIT score 20 or over) than women and non-residents. In the previous 4 weeks, 13% of students had been insulted or humiliated and 6% had been pushed, hit or otherwise assaulted by others who were drinking. One percent of respondents had experienced sexual assault in this time period.

**Conclusions:**

Half of men and over a third of women were drinking at hazardous levels and a relatively large proportion of students were negatively affected by their own and other students' drinking. There is a need for intervention to reduce hazardous drinking early in university participation.

**Trial registration:**

ACTRN12608000104358

## Background

A high prevalence of hazardous drinking by university students has been reported in many countries [1-3] with this population group often drinking more than their non-university/college student peers [4-7]. In large-scale national surveys in the United States, 37-44% of students report binge drinking (more than five standard drinks per occasion; each containing 12 g ethanol) in the previous two weeks [8-10] with men drinking more than women, although this difference has narrowed over time [10-12]. Among New Zealand (NZ) university students, 37% have reported one or more binge episodes in the previous week [13].

Factors within the university environment contribute to these high levels of consumption leading to a range of negative consequences [5,7,14]. These include: social, physical and psychological harms to the student e.g. academic impairment, blackouts, injury, suicide, unintended sexual activity and sexual coercion; harm to other people including interpersonal and sexual violence; and costs to the institution such as property damage and student attrition [13,15-21]. The secondhand effects of people's drinking on others are also assuming greater importance for advocacy in alcohol control policy, both for the victims experiencing assaults, sexual violence and impacts on studying [18,22] and for the wider community experiencing litter, noise and vandalism [23].

Australian studies report between 70-96% of university students regularly consume alcohol [24-29] with 50% drinking to intoxication at least weekly [30,31]. However, previous studies have relied on convenience samples [24-39] and most are at least a decade old [24-33,38]. The one Australian study that used a random sample [40] surveyed only international students and therefore is not generalizable to all university students. This study found, that 66% consumed alcohol and 2% drank five standard drinks or more per occasion once or more a week.

There is significant support for the use of the Internet to collect epidemiological data particularly among university populations [41-45]. Online surveys permit fast application and wide accessibility [46,47]. With their capacity for interactivity, automaticity, respondent anonymity and cost effectiveness [48-50], ability to facilitate more honest and thoughtful responses [42,51] and good validity and reliability [43,52-57], a carefully conducted online survey may help overcome many of the barriers associated with collecting epidemiological data [58-60]. Unlike the proportionate cost to attain large sample sizes using traditional modes of survey implementation, marginal costs are low and therefore they are advantageous for large sample sizes [59,61]. In addition, unique features such as complex logic and branching [62] and real-time error checking and automated data entry [63] allow statistical processing to occur in real time [64]. This enables web-survey technology to deliver concurrent feedback interventions [65]. It may be ethically obligatory when surveys identify harmful behaviours among respondents to provide feedback. This may be an efficient option given that provision of immediate feedback in this context has been shown to change behaviour [65,66].

As part of a larger efficacy trial of a web-based alcohol screening and brief intervention [65,67], this study estimated the frequency and quantity of alcohol consumption, and prevalence of hazardous drinking and secondhand effects among a large sample of undergraduate students attending a university in Perth, Western Australia.

## Methods

### Participants

A random sample of 13,000 undergraduate students aged 17-25 years, enrolled full-time and studying on campus at a Western Australian university, were invited to complete a web survey on alcohol consumption, secondhand effects, attitudes toward nutrition/ingredient labeling [68] and tobacco use [69]. Women made up 52.4% of the sample and 20.6% were non-residents. The term 'non-resident' refers to students enrolled at the university that are not permanent residents of Australia or New Zealand and includes those on student visas and humanitarian visas.

### Procedure

We adopted a survey recruitment procedure described in detail elsewhere [59,65,67,70]. Four weeks after the start of the first semester of 2007 the University Surveys Office accessed the enrolment database to identify a random sample of 13,000 full-time undergraduates aged 17-25 years. A personally addressed letter from the research team was sent to each student, inviting them to participate in the survey. The letter explained that they would soon receive a hyperlink to the questionnaire in an email message, that responses would be confidential and that the research team was independent of the university administration. Students were offered the opportunity to win one of 40 AU$100 gift vouchers for participating. After 1 week, a reminder email was sent encouraging completion of the questionnaire to those who had not yet responded. A second reminder was sent 10 days later.

### Measures

The questionnaire included items on: past alcohol use [71]; current alcohol use [Alcohol Use Disorders Identification Test (AUDIT) [72]]; peak consumption in the previous 4 weeks [73]; height and weight (in order to estimate Blood Alcohol Concentration); secondhand effects of drinking [22]; attitudes toward nutrition/ingredient labelling on alcohol packaging [68]; and tobacco use [74]. The use of standardised instruments for measuring personal use [Alcohol Use Disorders Identification Test (AUDIT) [72]] and secondhand effects [22] make it comparable to studies carried out in other countries. The complete wording and layout of all items can be seen at: http://lamp.health.curtin.edu.au/thrive/baselinetest.php.

### Data analysis plan

Descriptive statistics were computed for the following: demographic data [age (17-19, 20-25 year olds), sex, and citizenship (Australian and NZ residents, non-residents)] of respondents and the sample; early or late response to the survey; the quantity and frequency of alcohol use; AUDIT scores; and the number of secondhand effects. Three AUDIT subscale scores were calculated to measure alcohol consumption (AUDIT items 1-3), dependence (items 4-6) and problems (or adverse consequences) (items 7-10) [72,75]. Total AUDIT scores were divided into four ordinal categories: moderate (0-7), hazardous (8-15), harmful (16-19), and dependent (20-40) [72]; and binary categories of hazardous (≥ 8) and non-hazardous (< 8).

The representativeness of responders to the random sample was assessed using chi-squared tests. The association between participant demographics and being either early or late responder, or to having an AUDIT score ≥ 8, was assessed using chi-squared tests. T-tests were used to compare the mean AUDIT measure for the three subscales (alcohol consumption, dependence and problems) against participant age, sex, and citizenship and to compare total AUDIT score between early and late responders.

The association of secondhand effects experience to participant demographics and frequency of consuming six or more drinks on one occasion (item three in the AUDIT [72]) was also assessed using chi-squared tests. The association between frequencies of consuming six or more drinks (60 g ethanol) on secondhand effects was assessed using multivariable logistic regression after adjusting for gender, age and citizenship. Results are presented as odds-ratio and associated 99% confidence intervals.

The associations between age, sex and citizenship and hazardous drinking were analysed using binary logistic regression. To protect against small effects being considered as being statistically significant due to the large sample size in the study, *p*-values of < 0.01 will be considered as statistically significant. The assumptions behind the statistical models fitted were assessed to ensure validity of results.

This study received ethical approval from Curtin University [HR 189/2005] and is registered with the Australian and New Zealand Clinical Trial Register [ACTRN12608000104358].

## Results

Of the 13,000 students invited, 7,237 responded (56% response rate) with 57% of these being women (n = 4123) and 16.2% non-Australian/NZ residents (n = 1172). The mean age of the respondents was 19.5 years (SD = 1.9). There was a higher representation of women, Australian/NZ residents and those aged 17-19 years among the survey respondents compared to the sample (*p *< 0.001) (see Table 1). The small differences (less than 5%) between early (before the second reminder) and late (after the second reminder) survey responders in relation to age, citizenship and gender were not significant. There was also no significant difference in mean AUDIT score between early and late survey responders.

**Table 1:** Demographic comparison of responders vs. study sample and early vs. late responders

		Sample **N **= **13000**	Responders **N **= **7237**	*p*-value	Early responder **N **= **4627**	Late responder **N **= **2610**	*p*-value
**Demographic characteristic**		**% (N)**	**% (N)**		**% (N)**	**% (N)**	

**Age**	17-19	48.6 (6321)	54.7 (3956)	< 0.001	53.3 (2465)	57.1 (1491)	0.020
	
	20-25	51.4 (6679)	45.3 (3281)		46.7 (2162)	42.9 (1119)	

**Gender**	Female	52.4 (6811)	57.0 (4123)	< 0.001	57.5 (2660)	56.1 (1463)	0.024
	
	Male	47.6 (6189)	43.0 (3114)		42.5 (1967)	43.9 (1147)	

**Citizenship**	Aust/NZ	79.4 (10322)	83.8 (6065)	< 0.001	84.5 (3909)	82.6 (2156)	0.370
	
	Non-Aust/NZ	20.6 (2678)	16.2 (1172)		15.5 (718)	17.4 (454)	

### Consumption characteristics

Ninety percent of respondents had consumed alcohol in the last 12 months, with a mean volume per typical occasion of 5.1 (SD = 5.0) standard drinks for women and 8.7 (SD = 8.6) for men. The National Health and Medical Research Council (Australia) thresholds for acute harm (40 g/60 g ethanol for women/men) [76] were exceeded at least once in the last 4 weeks by 48% of respondents.

A significantly higher mean AUDIT score (*p *< 0.001) was observed for men (8.6; SD = 6.9) than women (6.5; SD = 5.9); and for Australian/NZ residents (8.1; SD = 6.4) than non-residents (3.5; SD = 4.6). There was not a significant difference (*p *= 0.730) between 17 and 19 year olds (7.7; SD = 6.4) and 20-25 year olds (7.0; SD = 6.5). There were significant differences in the proportions scoring 8 or higher on the AUDIT (see Table 2) with 44.5% of 17-19 year olds versus 39.1% of 20-25 year olds (*p *< 0.001), 50.6% of men versus 35.7% of women (*p *< 0.001) and 47.0% of residents versus 16.5% of non-residents (*p *< 0.001). Men and Australian/NZ residents had significantly increased odds (OR: 2.1; 95% CI: 1.9-2.3; OR: 5.2; 95% CI: 4.4-6.2) of being categorised as dependent (AUDIT score 20 or over) compared to women and non-residents.

**Table 2:** AUDIT subscale scores and hazardous drinking (AUDIT Score ≥ 8) by demographic characteristics

		AUDIT Subscales			AUDIT Score ≥ 8 % (N)
			
Demographic characteristic		AUDIT Consumption^a^ Mean (SD)	AUDIT Dependence^b^ Mean (SD)	AUDIT Problems^c^ Mean (SD)	
**Age**	17-19	4.8 (SD = 3.3)	0.8 (SD = 1.4)	2.1 (SD = 2.8)	44.5 (1762)
	
	20-25	4.3 (SD = 3.2)	0.8 (SD = 1.4)	1.9 (SD = 2.8)	39.1 (1284)
	
	*p*-value	< 0.001	0.587	0.35	< 0.001

**Gender**	Female	4.0 (SD = 3.0)	0.7 (SD = 1.2)	1.8 (SD = 2.6)	35.7 (1471)
	
	Male	5.3 (SD = 3.5)	1.0 (SD = 1.6)	2.3 (SD = 3.0)	50.6 (1575)
	
	*p*-value	< 0.001	< 0.001	< 0.001	< 0.001

**Citizenship**	Aust/NZ	5.0 (SD = 3.2)	0.9 (SD = 1.4)	2.3 (SD = 2.9)	47.0 (2853)
	
	Non-Aust/NZ	2.3 (SD = 2.6)	0.4 (SD = 1.0)	0.8 (SD = 1.9)	16.5 (193)
	
	*p*-value	< 0.001	< 0.001	< 0.001	< 0.001

^a ^Consists of AUDIT items 1-3: How often do you have a drink containing alcohol (0-4); How many drinks containing alcohol do you have on a typical day when you are drinking (0-4); and How often do you have six or more drinks no one occasion (0-4) [Total range 0-12]

^b ^Consists of AUDIT items 4-6: How often during the last year have you found that you were not able to stop drinking once you had started (0-4); How often during the last year have you failed to do what you normally expected from you because of drinking (0-4) and How often during the last year have you needed a first drink in the morning to get yourself going after a heavy drinking session (0-4) [Total range 0-12]

^c ^Consists of AUDIT items 7-10: How often during the last year have you had a feeling of guilt or remorse after drinking? (0-4); How often during the last year have you been unable to remember what happened the night before because you had been drinking? (0-4); Have you or someone else been injured as a result of your drinking? (0-4); Has a relative or friend or a doctor or another health worker been concerned about your drinking or suggested you cut down? (0-4) [Total range 0-16]

Men had higher odds of drinking at hazardous levels compared to women (OR: 2.0; 95% CI: 1.8-2.2). Australian/NZ residents had higher odds compared to non-residents (OR: 5.1; 95% CI: 4.3-6.0) and the association with age was non-significant (*p *= 0.113).

Significantly higher mean AUDIT scores (*p *< 0.001) were observed for men and Australian/NZ residents compared to women and non-residents in all AUDIT subscales (shown in Table 2). There were significant differences in relation to age in the AUDIT Consumption subscale with higher mean scores for 17-19 year olds compared to 20-24 year olds, but not the other subscales.

### Secondhand effects

The 4-week prevalence of secondhand effects is shown in Table 3. The most commonly reported effects were having to 'baby-sit' inebriated students (27.2%); having studying or sleep interrupted (20.9%); being insulted or humiliated (12.9%); having a serious argument (12.5%); or experiencing an unwanted sexual advance (10.9%).

**Table 3:** Secondhand effects experience by demographic characteristics

Secondhand effects	Age			Gender			Citizenship		
	
	17-19	20-25	*p*-value	Female	Male	*P*-value	Aust/NZ	Non	*p*-value
						
	% (N)	% (N)		% (N)	% (N)		% (N)	% (N)	
Been insulted or humiliated	13.6 (535)	12.1 (396)	0.070	12.2 (500)	13.9 (431)	0.027	13.4 (207)	10.7 (124)	0.015

Had a serious argument or quarrel	13.7 (541)	11.1 (362)	0.001	12.6 (518)	12.4 (385)	0.808	13.2 (794)	9.4 (109)	< 0.001

Been pushed, hit or otherwise assaulted	7.2 (283)	5.6 (181)	0.005	4.8 (195)	8.7 (269)	< 0.001	6.7 (403)	5.3 (61)	0.074

Had your property damaged	8.3 (325)	7.0 (227)	0.039	7.2 (296)	8.3 (256)	0.098	7.5 (452)	8.6 (100)	0.185

Had to 'baby-sit' or take care of another student who had drunk too much	31.9 (1256)	21.6 (706)	< 0.001	28.8 (1184)	25.1 (778)	0.001	28.8 (1742)	19.0 (220)	< 0.001

Found vomit in the halls or bathroom of your residence	6.5 (257)	6.0 (196)	0.364	5.6 (231)	7.2 (222)	0.008	5.6 (337)	10.0 (116)	< 0.001

Had your studying or sleep interrupted	22.1 (872)	19.4 (633)	0.004	21.5 (884)	20.1 (621)	0.128	20.1 (1215)	25.0 (290)	< 0.001

Experienced unwanted sexual advances	12.1 (474)	9.5 (310)	0.001	13.8 (565)	7.1 (219)	< 0.001	11.9 (717)	5.8 (784)	< 0.001

Been a victim of sexual assault	0.9 (35)	1.1 (36)	0.358	0.9 (36)	1.1 (35)	0.289	0.8 (47)	2.1 (24)	< 0.001

Been a victim of another crime on campus	0.8 (30)	1.1 (35)	0.165	0.8 (34)	1.0 (31)	0.448	0.6 (39)	2.2 (26)	< 0.001

Been a victim of another crime off campus	2.4 (93)	2.0 (66)	0.328	1.8 (74)	2.8 (85)	0.007	2.1 (125)	2.9 (34)	0.067

Men were more likely than women to experience being 'pushed, hit or otherwise assaulted' (8.7% vs. 4.8%; *p *< 0.001) and to have been a victim of another crime off campus (2.8% vs. 1.8%; *p *= 0.007) while women were more likely to experience an unwanted sexual advance (13.8% vs. 7.1%; *p *< 0.001) and to have had to 'baby-sit' or take care of another student who had too much to drink (28.8% vs. 25.1%; *p *= 0.001). Those aged 17-19 years were more likely than 20-25 year olds to have had a serious argument (13.7% vs. 11.1%; *p *= 0.001); been assaulted (7.2% vs. 5.6%; *p *= 0.005); had to 'baby-sit' another student (31.9% vs. 21.6%; *p *< 0.001); had their studying or sleep interrupted (22.1% vs. 19.4%; *p *= 0.004) or to have experienced unwanted sexual advances (12.1% vs. 9.5%; *p *= 0.001).

There was a significant difference based on citizenship for most secondhand effects with Australian/NZ residents more likely than non-residents to have had a serious argument or quarrel (13.2% vs. 9.4%; *p *< 0.001); had to baby-sit another student (28.8% vs. 19.0%; *p *< 0.001) or to have experienced an unwanted sexual advance (11.9% vs. 5.8%; *p *< 0.001). Non-residents on the other hand were more likely to have had their studying or sleep interrupted (25.0% vs. 20.1%; *p *< 0.001); been a victim of sexual assault (2.1% vs. 0.8%); been a victim of another crime on campus (2.2% vs. 0.6%; *p *< 0.001) and were almost twice as likely to have found vomit in the halls or bathroom of their residence (10.0% vs. 5.6%; *p *< 0.001). The odds of experiencing most secondhand effects increases with increasing frequency of consuming six or more drinks (60 g ethanol) on one occasion, after adjusting for gender, age and citizenship. Being a victim of sexual assault, and being a victim of another crime on and off campus are not significantly associated with the frequency of this level of alcohol consumption (see Table 4).

**Table 4:** Effects* of frequency of consuming six or more drinks (60 g ethanol) on secondhand effects

	Frequency of consuming six or more drinks (cf Never)		
	
Secondhand effect	Less than monthly OR (99% CI)	2-4 times a month OR (99% CI)	> 2 times a week OR (99% CI)
Been insulted or humiliated	1.39 (1.03-1.90)	1.88 (1.35-2.62)	2.57 (1.88-3.51)

Had a serious argument or quarrel	1.95 (1.36-2.79)	3.15 (2.16-4.60)	5.97 (4.19-8.52)

Been pushed, hit or otherwise assaulted	1.18 (0.76-1.85)	1.54 (0.95-2.49)	3.02 (1.96-4.64)

Had your property damaged	1.66 (1.11-2.47)	2.23 (1.45-3.44)	2.68 (1.77-4.05)

Had to 'baby-sit' or take care of another student who had drunk too much	1.96 (1.56-2.47)	2.81 (2.18-3.62)	3.66 (2.87-4.66)

Found vomit in the halls or bathroom of your residence	1.51 (0.97-2.34)	2.50 (1.56-4.01)	3.58 (2.29-5.60)

Had your studying or sleep interrupted	1.44 (1.12-1.86)	2.32 (1.77-3.06)	3.26 (2.51-4.23)

Experienced unwanted sexual advances	2.11 (1.46-3.05)	2.86 (1.93-4.24)	5.38 (3.72-7.79)

Been a victim of sexual assault (this includes date rape)	0.79 (0.31-1.97)	0.88 (0.29-2.69)	1.62 (0.63-4.17)

Been a victim of another crime on campus	0.65 (0.24-1.76)	0.77 (0.22-2.63)	1.56 (0.57-4.26)

Been a victim of another crime off campus	0.96 (0.48-1.89)	1.33 (0.63-2.79)	1.74 (0.88-3.45)

*Results are presented as odds-ratio and associated 99% confidence intervals after adjusting for gender, age and citizenship in multivariable logistic regression

## Discussion

This study is the first known prevalence study of student drinking completed in Australia with undergraduate students. The vast majority of students were current drinkers (90%) and there was a high prevalence of hazardous drinking (48%), with a higher prevalence among men compared with women, and in Australian/NZ residents compared with non-residents. A relatively large proportion of students' experienced secondhand effects from other people's drinking.

The survey had a response rate of 56%, which is higher than large college surveys in the early 2000s in the United States (52%) [10], but lower than online surveys in New Zealand using similar procedures (63-82%) [13,59]. Higher response rates for online surveys have been linked to pre-notification, personalised contacts and follow up reminders [77]. Both this and the New Zealand studies incorporated pre-notification, personalised emails and follow-up notices. However, the earlier New Zealand study used up to nine follow-up contacts (compared to five in this study) including a telephone reminder, which may explain some of the difference. Follow-up notices are likely to increase response rates though larger numbers of notices may not appreciably affect response if the contact develops resistance to participation [78]. It is also possible that the novelty factor of online surveys may have reduced in the years since the New Zealand studies and factors such as proliferation of junk mail, bombardment with online questionnaires and demands on student time may also have impacted on response rates [47].

The level of alcohol consumption reported in this study is less than that reported in New Zealand, for both men and women [13]. Although gender convergence in drinking has been reported elsewhere [10-12,79] and a similar trend appears to be occurring in Australia [80], this study shows a large discrepancy between men and women. However, there are no older prevalence studies from which to assess attenuation trends.

Large numbers of people were affected by other students' drinking. Of particular note was the 0.9% (n = 36) of women and 1.1% (n = 35) of men who reported being a victim of sexual assault in the previous 4 weeks. This is slightly higher than that found in a New Zealand sample [81] though with overlapping confidence intervals. While the New Zealand sample was limited to those who had consumed alcohol in the previous 4 weeks, our sample included non-heavy drinkers and may highlight the impact that hazardous alcohol consumption can have on all students. Extrapolated to the entire student population this may mean approximately 140 students at this university experience sexual assault in this context each month.

A limitation of this study was the imprecision in the specificity of crimes listed in the secondhand effects questions and the reliance on respondents to attribute responsibility for the effect. As only yes or no responses were available, multiple experiences of the same effect were not captured and therefore the prevalence of these effects may be underestimated. Given the high prevalence of some of these effects further research in this area is warranted. Our estimates may be biased by selective non-response but conversely computerised questionnaires are known to increase reporting of high-risk behaviour [42,51].

Universities with large on-campus resident populations may have higher levels of drinking than commuter universities due to students' greater proximity to peers [82]. As this study is based on a single commuter university and has a high proportion of students on temporary visas, the findings may be limited in their generalisability.

This study highlights the need for university programs to target drinking in this population. With half of male, and over a third of female, respondents drinking at hazardous levels, population approaches are needed. The literature suggests that programs should also address environmental factors, particularly the availability and promotion of alcohol on and around campus [22,83].

## Conclusions

Hazardous alcohol use among undergraduate students remains an issue of concern although there is a lack of prevalence data on this population's alcohol consumption in Australia. Some alcohol related harms such as sexual assault are only detected with large population samples. Web-based surveys are a cost-effective approach for measuring health behaviours in student populations, with a relatively high response rate. It is suggested that this research is replicated in other Australian universities, particularly residential campuses. Such surveys are required to develop trend data which will facilitate intervention program development.

## Competing interests

The authors declare that they have no competing interests.

## Authors' contributions

JH carried out the study and drafted the manuscript. PH, AM, KK and BM conceived of the study and participated in its design and coordination. SD participated in the design of the study and performed the statistical analysis. PH, BM, KK and AM helped to draft the manuscript. All authors read and approved the final manuscript.

## Pre-publication history

The pre-publication history for this paper can be accessed here:

http://www.biomedcentral.com/1471-2458/12/37/prepub

## References

[B1] Karam E Kypri K Salamoun M Alcohol use among college students: an international perspective Curr Opin Psychiatr 2007 20 213 221 10.1097/YCO.0b013e3280fa836c 17415072

[B2] Wechsler H Nelson TF What we have learned from the Harvard School Of Public Health College Alcohol Study: focusing attention on college student alcohol consumption and the environmental conditions that promote it J Stud Alcohol Drugs 2008 69 481 490 18612562 10.15288/jsad.2008.69.481

[B3] Wicki M Kuntsche E Gmel G Drinking at European universities? A review of students' alcohol use Addict Behav 2010 35 913 924 10.1016/j.addbeh.2010.06.015 20624671

[B4] Dawson DA Grant BF Stinson FS Chou PS Another look at heavy episodic drinking and alcohol use disorders among college and noncollege youth J Stud Alcohol 2004 65 477 15378804 10.15288/jsa.2004.65.477

[B5] Kypri K Cronin M Wright CS Do university students drink more hazardously than their non-student peers? Addiction 2005 100 713 714 10.1111/j.1360-0443.2005.01116.x 15847629

[B6] Reifman A Ro H Barnes GM Feng D Drinking in Youth Ages 13-21 attending and not attending college J First Year Exp Stud Trans 2010 22 67 86

[B7] Slutske WS Hunt-Carter EE Nabors-Oberg RE Sher KJ Bucholz KK Madden PA Anokhin A Heath AC Do college students drink more than their non-college-attending peers? Evidence from a population-based longitudinal female twin study J Abnorm Psychol 2004 113 530 540 15535786 10.1037/0021-843X.113.4.530

[B8] Johnston LD O'Malley PM Bachman JG Schulenberg JE Monitoring the Future national survey results on drug use, 1975-2009: Volume II, College students and adults ages 19-50 2010 Bethesda, MD: National Institute on Drug Use

[B9] Saltz RF Welker LR Paschall MJ Feeney MA Fabiano PM Evaluating a comprehensive campus-community prevention intervention to reduce alcohol-related problems in a college population J Stud Alcohol Drugs Suppl 2009 16 21 27 19538909 10.15288/jsads.2009.s16.21 PMC2701100

[B10] Wechsler H Lee JE Kuo M Seibring M Nelson TF Lee H Trends in college binge drinking during a period of increased prevention efforts. Findings from 4 Harvard School of Public Health College Alcohol Study surveys: 1993-2001 J Am Coll Health 2002 50 203 217 10.1080/07448480209595713 11990979

[B11] Keyes KM Grant BF Hasin DS Evidence for a closing gender gap in alcohol use, abuse, and dependence in the United States population Drug Alcohol Depend 2008 93 21 29 10.1016/j.drugalcdep.2007.08.017 17980512 PMC3163892

[B12] Kuntsche E Kuntsche S Knibbe R Simons-Morton B Farhat T Hublet A Bendtsen P Godeau E Demetrovics Z Cultural and gender convergence in adolescent drunkenness: evidence from 23 European and North American countries Arch Pediatr Adolesc Med 2011 165 152 158 10.1001/archpediatrics.2010.191 20921343 PMC4133118

[B13] Kypri K Paschall MJ Langley J Baxter J Cashell-Smith M Bourdeau B Drinking and alcohol-related harm among New Zealand university students: findings from a national web-based survey Alcohol Clin Exp Res 2009 33 307 314 10.1111/j.1530-0277.2008.00834.x 19032577

[B14] Weitzman ER Nelson TF Wechsler H Taking up binge drinking in college: the influences of person, social group, and environment J Adolesc Health 2003 32 26 35 10.1016/S1054-139X(02)00457-3 12507798

[B15] Lewis MA Rees M Logan DE Kaysen DL Kilmer JR Use of drinking protective behavioral strategies in association to sex-related alcohol negative consequences: the mediating role of alcohol consumption Psychol Addict Behav 2010 24 229 238 20565149 10.1037/a0018361 PMC2891544

[B16] Mallett KA Bachrach RL Turrisi R Are all negative consequences truly negative? Assessing variations among college students' perceptions of alcohol related consequences Addict Behav 2008 33 1375 1381 10.1016/j.addbeh.2008.06.014 18639384 PMC2566513

[B17] Murphy JG Hoyme CK Colby SM Borsari B Alcohol consumption, alcohol-related problems, and quality of life among college students J Coll Student Dev 2006 47 110 121 10.1353/csd.2006.0010

[B18] Nelson TF Xuan Z Lee H Weitzman ER Wechsler H Persistence of heavy drinking and ensuing consequences at heavy drinking colleges J Stud Alcohol Drugs 2009 70 726 734 19737497 10.15288/jsad.2009.70.726

[B19] Park CL Positive and negative consequences of alcohol consumption in college students Addict Behav 2005 29 311 321 14732419 10.1016/j.addbeh.2003.08.006

[B20] Perkins HW Surveying the damage: a review of research on consequences of alcohol misuse in college populations J Stud Alcohol Suppl 2002 14 91 100 12022733 10.15288/jsas.2002.s14.91

[B21] Ray AE Turrisi R Abar B Peters KE Social-cognitive correlates of protective drinking behaviors and alcohol-related consequences in college students Addict Behav 2009 34 911 917 10.1016/j.addbeh.2009.05.016 19540676 PMC2842573

[B22] Langley JD Kypri K Stephenson SC Secondhand effects of alcohol use among university students: computerised survey Brit Med J 2003 327 1023 1024 10.1136/bmj.327.7422.1023 14593036 PMC261656

[B23] Wechsler H Lee JE Hall J Wagenaar AC Lee H Secondhand effects of student alcohol use reported by neighbors of colleges: the role of alcohol outlets Soc Sci Med 2002 55 425 435 10.1016/S0277-9536(01)00259-3 12144150

[B24] Adams WG Survey of tobacco and alcohol use among undergraduates Med J Australia 1979 2 160

[B25] Engs RC Drinking patterns and attitudes toward alcoholism of Australian human-service students J Stud Alcohol 1982 43 517 531 7144183 10.15288/jsa.1982.43.517

[B26] Neil JV Student drinking patterns: their implications for health education programmes Forum Educ 1978 37 22 26

[B27] O'Callaghan F Callan VJ Wilks J Extending upon student drinking patterns in an Australian population Drug Alcohol Rev 1990 9 239 244 10.1080/09595239000185311 16840145

[B28] Sargent M Drinking and alcoholism in Australia: a power relations theory 1979 Melbourne: Longman Cheshire

[B29] Wilks J Student drinking patterns: experience in an Australian population Aust Drug Alcohol Rev 1986 5 3 7 10.1080/09595238680000021 16840145

[B30] Isralowitz R Borowski A Ong TH Male and female differences in alcohol use patterns and behavior J Alcohol Drug Educ 1993 38 120 125

[B31] Roche A Watt K Drinking and university students: from celebration to inebriation Drug Alcohol Rev 1999 18 389 399 10.1080/09595239996257

[B32] Basten CJ Psychol M Kavanagh DJ Alcohol consumption by undergraduate students Subst Use Misuse 1996 31 1379 1399 10.3109/10826089609063982 8879079

[B33] Crundall IA Perceptions of alcohol by student drinkers at university Drug Alcohol Rev 1995 14 363 368 10.1080/09595239500185491 16203335

[B34] Davey JD Davey T Obst P Alcohol consumption and drug use in a sample of Australian university students Youth Stud Aust 2002 21 25 32

[B35] Davey JD Davey T Obst PL Drug and drink driving by university students: an exploration of the influence of attitudes Traffic Inj Prev 2005 6 44 52 10.1080/15389580590903168 15823874

[B36] Polizzotto MN Saw MM Tjhung I Chua EH Stockwell TR Fluid skills: drinking games and alcohol consumption among Australian university students Drug Alcohol Rev 2007 26 469 475 10.1080/09595230701494374 17701509

[B37] Reavley NJ Jorm AF McCann TV Lubman DI Alcohol consumption in tertiary education students BMC Public Health 2011 11 545 10.1186/1471-2458-11-545 21740593 PMC3223920

[B38] Stevenson M Palamara P Rooke M Richardson K Baker M Baumwol J Drink and drug driving: what's the skipper up to? Aust NZ J Publ Heal 2001 25 511 513 10.1111/j.1467-842X.2001.tb00314.x 11824985

[B39] Utpala-Kumar R Deane FP Rates of alcohol consumption and risk status among Australian university students vary by assessment questions Drug Alcohol Rev 2010 29 28 34 20078679 10.1111/j.1465-3362.2009.00082.x

[B40] Rosenthal RA Russell J Thomson G The health and wellbeing of international students at an Australian university Res High Educ 2008 55 51 67

[B41] Carini RM Hayek JC Kuh GD Kennedy JM Ouimet JA College Student responses to web and paper surveys: does mode matter? Res High Educ 2003 44 1 19 10.1023/A:1021363527731

[B42] Daley EM McDermott RJ McCormack Brown KR Kittleson MJ Conducting web-based survey research: a lesson in internet designs Am J Health Behav 2003 27 116 124 12639069 10.5993/ajhb.27.2.3

[B43] McCabe SE Boyd CJ Couper MP Crawford S D'Arcy H Mode effects for collecting alcohol and other drug use data: web and U.S. mail J Stud Alcohol 2002 63 755 761 12529076 10.15288/jsa.2002.63.755

[B44] McCabe SE Couper MP Cranford JA Boyd CJ Comparison of web and mail surveys for studying secondary consequences associated with substance use: evidence for minimal mode effects Addict Behav 2006 31 162 168 10.1016/j.addbeh.2005.04.018 15916862

[B45] McCabe SE Diez A Boyd CJ Nelson TF Weitzman ER Comparing web and mail responses in a mixed mode survey in college alcohol use research Addict Behav 2006 31 1619 1627 10.1016/j.addbeh.2005.12.009 16460882 PMC3156492

[B46] Norton TR Lazev AB Schnoll RA Miller SM The impact of email recruitment on our understanding of college smoking Addict Behav 2009 34 531 535 10.1016/j.addbeh.2009.03.017 19395180 PMC2802216

[B47] Sax LJ Gilmartin SK Bryant AN Assessing response rates and nonresponse bias in web and paper surveys Res High Educ 2003 44 409 432 10.1023/A:1024232915870

[B48] Dillman DA Mail and Internet Surveys: The Tailored Design Method 2007 2 New Jersey: John Wiley & Sons, Inc.

[B49] Eysenbach G Wyatt J Using the Internet for surveys and health research J Med Internet Res 2002 4 E13 10.2196/jmir.4.2.e13 12554560 PMC1761932

[B50] Sills SJ Song C Innovations in survey research: An application of web-based surveys Soc Sci Comput Rev 2002 20 22 30 10.1177/089443930202000103

[B51] Turner CF Ku L Rogers SM Lindberg LD Pleck JH Sonenstein FL Adolescent sexual behavior, drug use, and violence: increased reporting with computer survey technology Science 1998 280 867 873 10.1126/science.280.5365.867 9572724

[B52] Cooper CJ Cooper SP del Junco DJ Shipp EM Whitworth R Cooper SR Web-based data collection: detailed methods of a questionnaire and data gathering tool Epidemiol Perspect Innov 2006 3 1 10.1186/1742-5573-3-1 16390556 PMC1343557

[B53] Couper MP Issues of representation in eHealth research (with a focus on Web surveys) Am J Prev Med 2007 32 S83 S89 10.1016/j.amepre.2007.01.017 17466823

[B54] Denscombe M Web-based questionnaires and the mode effect: an evaluation based on completion rates and data contents of near-identical questionnaires delivered in different modes Soc Sci Comput Rev 2006 24 246 254 10.1177/0894439305284522

[B55] Gosling SD Vazire S Srivastava S John OP Should we trust web-based studies? A comparative analysis of six preconceptions about internet questionnaires Am Psychol 2004 59 93 104 14992636 10.1037/0003-066X.59.2.93

[B56] Miller ET Neal DJ Roberts LJ Baer JS Cressler SO Metrik J Marlatt GA Test-retest reliability of alcohol measures: is there a difference between internet-based assessment and traditional methods? Psychol Addict Behav 2002 16 56 63 11934087

[B57] Schonlau M Will Web Surveys Ever Become Part of Mainstream Research? Journal of Medical Internet Research 2004 6 E31 10.2196/jmir.6.3.e31 15471757 PMC1550607

[B58] Bendtsen P Johansson K Akerlind I Feasibility of an email-based electronic screening and brief intervention (e-SBI) to college students in Sweden Addict Behav 2006 31 777 787 10.1016/j.addbeh.2005.06.002 15996827

[B59] Kypri K Gallagher SJ Cashell-Smith ML An internet-based survey method for college student drinking research Drug Alcohol Depend 2004 76 45 53 10.1016/j.drugalcdep.2004.04.001 15380288

[B60] McAlaney J McMahon J Normative beliefs, misperceptions, and heavy episodic drinking in a british student sample J Stud Alcohol Drugs 2007 68 385 392 17446978 10.15288/jsad.2007.68.385

[B61] Ekman A Litton JE New times, new needs; e-epidemiology Eur J Epidemiol 2007 22 285 292 10.1007/s10654-007-9119-0 17505896

[B62] Couper MP Web Survey Design and Administration Public Opin Q 2001 65 230 253 10.1086/322199 11420757

[B63] Solomon DJ Conducting web-based surveys Prac Assess Res Eval 2001 7 19

[B64] Baer A Saroiu S Koutsky LA Obtaining sensitive data through the Web: an example of design and methods Epidemiology 2002 13 640 645 10.1097/00001648-200211000-00007 12410004

[B65] Kypri K Hallett J Howat P McManus A Maycock B Bowe S Horton NJ Randomized controlled trial of proactive web-based alcohol screening and brief intervention for university students Arch Intern Med 2009 169 1508 1514 10.1001/archinternmed.2009.249 19752409

[B66] Kypri K Langley JD Saunders JB Cashell-Smith ML Herbison P Randomized controlled trial of web-based alcohol screening and brief intervention in primary care Arch Intern Med 2008 168 530 536 10.1001/archinternmed.2007.109 18332300

[B67] Hallett J Maycock B Kypri K Howat P McManus A Development of a web-based alcohol intervention for university students: processes and challenges Drug Alcohol Rev 2009 28 31 39 10.1111/j.1465-3362.2008.00008.x 19320673

[B68] Kypri K McManus A Howat PM Maycock BR Hallett JD Chikritzhs TN Ingredient and nutrition information labelling of alcoholic beverages: do consumers want it? Med J Aust 2007 187 669 18072914 10.5694/j.1326-5377.2007.tb01469.x

[B69] Howat P Hallett J Kypri K Maycock B Dhaliwal S McManus A Tobacco smoking in an Australian university sample and implications for health promotion Prev Med 2010 51 425 426 10.1016/j.ypmed.2010.08.015 20817020

[B70] Kypri K Stephenson S Langley J Assessment of nonresponse bias in an internet survey of alcohol use Alcohol Clin Exp Res 2004 28 630 634 10.1097/01.ALC.0000121654.99277.26 15100615

[B71] AIHW 2004 National Drug Strategy Household Survey: detailed findings 2005 Cat. no. PHE 66: Canberra: AIHW

[B72] Saunders JB Aasland OG Babor TF de la Fuente JR Grant M Development of the Alcohol Use Disorders Identification Test (AUDIT): WHO collaborative project on early detection of persons with harmful alcohol consumption-II Addiction 1993 88 791 804 10.1111/j.1360-0443.1993.tb02093.x 8329970

[B73] Kypri K Langley J Stephenson S Episode-centred analysis of drinking to intoxication in university students Alcohol Alcohol 2005 40 447 452 15996969 10.1093/alcalc/agh178

[B74] Kypri K Baxter J Smoking in a New Zealand university student sample N Z Med J 2004 117 U794 15107897

[B75] O'Hare T Sherrer MV Validating the alcohol use disorder identification test with college first-offenders J Subst Abuse Treat 1999 17 113 119 10.1016/S0740-5472(98)00063-4 10435259

[B76] NHMRC Australian Alcohol Guidelines: Health Risks and Benefits 2001 Canberra: Commonwealth of Australia

[B77] Cook C Heath F Thompson RL A meta-analysis of response rates in web- or internet-based surveys Educ Psychol Meas 2000 60 821 836 10.1177/00131640021970934

[B78] Kittleson MJ Determining effective follow-up of e-mail surveys Am J Health Behav 1997 21 193 196

[B79] Wallace JM Jr Bachman JG O'Malley PM Schulenberg JE Cooper SM Johnston LD Gender and ethnic differences in smoking, drinking and illicit drug use among American 8th, 10th and 12th grade students, 1976-2000 Addiction 2003 98 225 234 10.1046/j.1360-0443.2003.00282.x 12534428

[B80] Roche AM Deehan A Women's alcohol consumption: emerging patterns, problems and public health implications Drug Alcohol Rev 2002 21 169 178 10.1080/09595230220139073 12188996

[B81] Connor J Gray A Kypri K Drinking history, current drinking and problematic sexual experiences among university students Aust N Z J Public Health 2010 34 487 494 10.1111/j.1753-6405.2010.00595.x 21040177

[B82] Kremer M Levy DM Peer effects and alcohol use among college students J Econ Perspect 2008 22 189 206 10.1257/jep.22.3.189

[B83] Howat P Sleet D Elder R Maycock B Preventing alcohol-related traffic injury: a health promotion approach Traffic Inj Prev 2004 5 208 219 10.1080/15389580490465238 15276921

